# Formation, Occurrence and Mitigation Strategies of Food Contaminants and Natural Toxicants: Challenges and Prospects

**DOI:** 10.3390/foods13040617

**Published:** 2024-02-19

**Authors:** Song Yu

**Affiliations:** Division of Chemical Toxicity and Safety Assessment, Shanghai Municipal Center for Disease Control and Prevention, Shanghai 200336, China; yusong@scdc.sh.cn; Tel.: +86-21-62758710

Food safety issues arising from food contaminants are one of the major challenges to global public health [1]. In order to prevent and mitigate hazardous exposures to food contaminants, it is important to determine the sources and resulting health problems at the time of exposure [2]. The development of methods to identify food contaminants and the investigation of their formation mechanisms are essential for effective regulation, the control of contaminants, and the reduction in their impact on human health [3]. This Special Issue covers these topics, including articles on the mechanisms of the formation of chemical contaminants in food production and the potential health problems associated with exposure to these contaminants, as well as naturally existing contaminants in food. These topics align with the third goal of the United Nations Sustainable Development Goals (SDGs), which aims to significantly reduce the disease burden caused by chemical contamination by 2030 [4]. These studies provide new ideas for the control of food contaminants.

Lai et al. explored the effects of oils and processing conditions on the formation of heterocyclic amines (HAs) and polycyclic aromatic hydrocarbons (PAHs) in pork fiber. The total HAs produced by frying pork fiber at 160 °C for 15 min were higher than those produced by frying at 200 °C for 6 min, with sesame oil or lard as the frying medium. However, under the same heating conditions, the levels of total HAs and total PAHs were higher in the pork fibers fried with sesame oil than with lard. In addition, the highest total PAH content was found in pork fiber fried in lard at 200 °C/6 min, followed by pork fiber fried in sesame oil at 200 °C/6 min and 160 °C/15 min and pork fiber fried in lard at 160 °C/15 min. In order to speculate on the formation mechanism of HA and PAH in fried pork fiber, their precursors were also determined. Principal component analysis also revealed the different formation mechanisms of HAs and PAHs in fried pork fiber.

Zheng et al. investigated whether the arsenic (As) content, speciation, and toxicity in germinated rice could be alleviated by selenium. The content and form of arsenic in 38 rice samples from two provinces were analyzed in the study. The method of reducing the risk of arsenic poisoning using biofortified selenium was investigated in germinated rice. According to the analysis of different parts of rice samples, the content of total As in the milled rice samples ranged from 0.03 to 0.37 mg/kg, and the average value of As (III) was almost twice that of As (V) in the milled rice and brown rice, with obvious regional differences. Co-exposure of arsenic and selenium in germinated rice showed a significant reduction in the accumulation of total As, As (III), and As (V), especially in milled rice. As (III) was oxidized to As (V) by the OH radical in the germinating rice. Therefore, the data showed that the addition of selenium has the potential to reduce the risk of arsenic poisoning in rice and have a beneficial effect.

Yu et al. examined the individual and combined cytotoxic effects of co-occurring fumonisin (FBs) family mycotoxins on porcine intestinal epithelial cells. In this study, the porcine intestinal epithelial cell was used as an in vitro model to determine the toxicity of fumonisin B1 (FB1), fumonisin B2 (FB2), and fumonisin B3 (FB3). They found that FBs significantly reduced cell viability, and the toxicity level was FB1 > FB2 > FB3. The interaction of the fumonisin combination is either synergistic or antagonistic, depending on the concentration. In addition, myriocin and resveratrol may reduce the cytotoxicity of FBs on IPEC cells. Finally, this result helps to determine the acceptable range of fumonisin in the feed and food industry. In particular, further attention and evaluation of the synergistic effects of low-concentration toxins are needed. A potential way to manage the toxicity of FBs was provided.

Squeo et al. discovered acrylamide contamination in plant-based protein ingredients. This contamination varied widely between natural pulse flour, protein concentrate, and textured protein. The evidence suggests that these ingredients and related plant-based foods are of concern. Key stages in the treatment process should be identified and appropriate mitigation strategies applied. Finally, given the increasing demand for and utilization of plant-based protein ingredients, it is necessary for policy makers and regulators to consider the monitoring and regulating of these ingredients.

Suryoprabowo et al. developed a highly sensitive monoclonal antibody for the detection of imidacloprid. The proposed lateral flow assay is a rapid and sensitive method for the detection of imidacloprid residues in fruits and vegetables with a cut-off value of 20 ng/mL. According to the recovery rate and CV value of the added samples, the method is rapid, sensitive, reliable, economical, and simple and can be used for the field detection of IMP residues in fruits and vegetables.

Albaridi et al. explored an approach to enhance the safety of ready-to-eat rice and extend the refrigerated preservation. Their results suggest that chitosan nanoparticles encapsulate the aromatic components of spices, preventing their biological activity from degrading during cooking. The water extract of the spice is known as the phenolic biological activity. The application of mixed extract types in a simulated fungal medium demonstrated the efficacy of aromatic extract in the production of anti-citrinin. In addition, the extracts extended the shelf life of ready-to-eat rice samples under both storage conditions (room temperature and refrigerated storage). These results suggest the application of an encapsulated aromatic extract obtained from ginger–thyme–coriander (at a ratio of 1:2:1) as a better treatment for extending the shelf life of ready-to-eat rice.

Negoita et al. investigated the effect of a number of pre-treatment applications on reducing acrylamide in frying potatoes under domestic conditions modeled on those found in Romania, using pans and frying pans. By pre-treating the potatoes before frying, such as soaking the potato strips in cold/hot water and soaking the potato strips in NaCl and citric acid solutions, the acrylamide content was reduced by 4–97% compared to the untreated control samples. Applying the strategies to reduce the acrylamide content in French fry samples was found to be effective. The effects of four kinds of pretreatment on the content of acrylamide in potato chips were as follows: cold water 42–89%, hot water 4–97%, NaCl solution 57–61%, citric acid solution 77–97%. There was a strong correlation between the acrylamide content and the color parameters L* and a* in French fries. The lower the acrylamide content, the lighter the color of the fries and the lower the redness parameter value. Soaking potatoes in cold water increases the hardness of the fries, while soaking them in hot water makes the product softer. In general, pretreatment to reduce the acrylamide levels in French fries and foods must be carried out accordingly so that consumers can accept that their nutritional quality, safety, and sensory properties are not compromised.

AI-Subaie et al. reviewed recent findings on pyrrolizidine alkaloid extraction and analysis. The pyrrolizidine alkaloid is a natural secondary metabolite that is mainly produced in plants, bacteria, and fungi as part of a biodefense mechanism. This compound makes up the largest class of alkaloids and is produced in nearly 3% of flowering plants, most of which belong to the Asteraceae and borage families. Pyrrolizidine alkaloids are toxic to humans and mammals. Therefore, the ability to detect these alkaloids in foods and nutrients is a food security issue. In this paper, the latest progress in the extraction and analysis of these alkaloids was reviewed, with emphasis on the chromatographic analysis and determination of these alkaloids in food. Moreover, the problems and challenges with the analysis were forecasted.

With the development of science and technology, humankind’s pursuit of high-quality food, and the desire for a healthy life, researchers should focus more on the identification system of food contaminants, safety assessment, governmental regulatory measures, and environmentally friendly contamination control methods.

In terms of analytical methods, developing high-trace, high-precision, rapid, and widely applicable analytical methods for food contaminants is promising to improve the detection capabilities and reduce the probability of false-positive or false-negative results. At the same time, with help of non-targeted analytical techniques and artificial intelligence technologies, new contaminants with potential hazards can be identified, and a high-exposure list can be established.

In terms of safety assessment, the research has evolved from the traditional assessment of single pollutants to the joint hazard assessment of mixed pollutants and from the previous risk assessment of dietary exposure (external exposure assessment) to the risk assessment of exposure to pollutants in human biological samples (urine, blood) (internal exposure assessment). In order to achieve the goal of better protecting the health of sensitive populations, more attention should be paid to the risk of exposure for infants, the elderly, and individuals with underlying health conditions.

Regarding regulation, the government should establish a comprehensive food safety risk assessment system and food safety protection regulations [5]. A rapid alert system will enable timely communication and response to food safety situations involving contaminants. In addition, strong financial support and developing technology facilitate the implementation of food risk monitoring programs [4]. 

For contaminant control, researchers should focus on the study of efficient, safe, and non-polluting control methods, such as the development of contaminant biodegradation enzymes and phytochemicals [6,7], verifying the practicality of contamination control methods in real-world sample matrices.

## Data Availability

Not applicable.
